# Mutations associated with retinopathies alter mitogen-activated protein kinase-induced phosphorylation of neural retina leucine-zipper

**Published:** 2007-07-12

**Authors:** Prabodha Swain, Sandeep Kumar, Dharmesh Patel, Sushmita Richong, Pranav Oberoi, Madhumita Ghosh, Anand Swaroop

**Affiliations:** 1 Department of Cellular and Molecular Neuroscience, National Brain Research Center, Manesar, Haryana, India; 2 Departments of Ophthalmology & Visual Sciences and Human Genetics, University of Michigan, MI

## Abstract

**Purpose:**

Neural retina leucine-zipper (NRL), a member of the basic motif leucine zipper family of transcription factors, is preferentially expressed in rod photoreceptors of the mammalian retina. Mutations in NRL are associated with retinopathies; many of these are suggested to change phosphorylation status and alter NRL-mediated transactivation of rhodopsin promoter. The purpose of this study was to identify potential kinases responsible for the phosphorylation of NRL and determine if such kinase-dependent phosphorylation is altered in disease-associated NRL mutations.

**Methods:**

Metabolic labeling with ^33^P-orthophosphate was used to study phosphorylation of NRL in transfected COS-1 cells. NRL or NRL mutants were expressed as glutathione S-transferase (GST)-fusion proteins and used as substrate to screen various kinases by in vitro phosphorylation assays. CV-1 cells were co-transfected with rhodopsin promoter-reporter construct and expression plasmids, with or without specific mitogen-activated protein kinase (MAPK) inhibitors, to examine their effect on NRL-mediated transactivation. Expression of activated MAPKs in postnatal mice retina was determined by immunoblot analysis.

**Results:**

Metabolic labeling of NRL produces multiple phosphorylated protein bands in transfected COS-1 cells. Fewer but more intense radiolabeled bands are observed for NRL-S50T, -S50A, and -P51L mutants compared to wild-type NRL. We show that MAPK2 and p38 induce specific phosphorylation of NRL, but this pattern is altered in NRL mutants. Immunoblot analysis of extracts from developing mouse retina reveals enhanced expression of activated MAPK2 at postnatal day 0-3, concordant with the reported phosphorylation pattern of NRL in vivo. Inhibition of MAPK signaling pathways decreases NRL and CRX -mediated synergistic activation of rhodopsin promoter in transfected CV-1 cells.

**Conclusions:**

Our results suggest that multiple MAPKs can phosphorylate NRL and this phosphorylation pattern is altered by disease-associated NRL mutations. As inhibition of MAPK signaling pathways decreases NRL-mediated transactivation of rhodopsin promoter, we propose that phosphorylation changes associated with NRL mutations perturb gene expression in rods, leading to photoreceptor degeneration in retinopathies.

## Introduction

Retina, a part of the central nervous system, serves as an ideal model for elucidating molecular mechanisms underlying complex neural functions of brain. The rod and cone photoreceptors are sensory neurons that initiate a cascade of phototransduction events to process visual signals in the retina [1]. Neural retina leucine-zipper (NRL), a member of basic motif leucine zipper (bZIP) family of DNA binding proteins, is preferentially expressed in developing and mature-rod photoreceptors and pineal gland [2-5]. It interacts with cone-rod homeobox (CRX) and other transcriptional regulatory proteins to activate the expression of most, if not all, rod photoreceptor genes [6-8]. NRL is critical for the differentiation of rod photoreceptors; its loss leads to cone-only retina in mouse, whereas ectopic expression of NRL converts cones to rods [4,9,10]. Mutations in the human *NRL* gene are associated with autosomal dominant retinitis pigmentosa (adRP) and other retinopathies [11-14]. It has been suggested that disease-causing mutations alter the phosphorylation of NRL and consequently affect its transcriptional regulatory function [11,14,15]. However, the precise biochemical mechanism(s) underlying NRL phosphorylation and its effect on NRL activity have not been delineated.

NRL belongs to *Maf*-oncogene subfamily and shares structural homology with Maf A, B, and c-Maf [16]. Previous studies have identified mitogen-activated protein kinases (MAPK) that produce specific phosphorylation of Mafs and thus regulate transdifferentiation and stabilization of lens cells from neuroretina [16,17]. In patients with adRP, similar conserved phosphorylation sites are mutated in the NRL protein, suggesting potential alteration of MAPK dependent phosphorylation of the protein in vivo [11,16]. A majority of NRL mutations have been identified in the transactivation domain and shown to alter synergistic activation of the rhodopsin promoter in cultured cells [11,14,15]. Nonetheless, biochemical mechanism(s) linking mutation-induced changes in the phosphorylation of NRL to signaling pathways associated with NRL activity or NRL-mediated gene regulation have not yet been reported.

In the current study, we performed metabolic phosphorylation of NRL to determine how mutations in NRL alter the nature of phosphorylation and consequently its activity. We have identified two MAPKs that produce specific phosphorylation of NRL. In corroborative experiments, we demonstrate an increased level of activated MAPK2 in postnatal mouse retina. We also report that disease-causing mutations alter MAPK-dependent phosphorylation of NRL and affect transactivation of rhodopsin promoter in transfected cells. Our studies suggest a role of MAPK signaling pathways in influencing NRL function during photoreceptor differentiation in mammalian retina.

## Methods

### Chemicals and reagents

Reagents and chemicals were purchased from New England Biolabs (NEB; Boston, MA) or Roche Diagnostics (Munich, Germany). Phospho-MAPK2, phospho-p38, protein kinase C (PKC) antibodies were purchased from Santa Cruz Biotechnology (Santa Cruz, CA), and MAPK1/2 antibody was obtained from Sigma (St. Louis, MO). The kinase inhibitors, U0126 and SB203580, were procured from Promega, Madison, WI. Purified activated-MAPK2 was obtained from NEB, whereas activated p38a was a gift from Prof. A. Khattar, R R Laboratories (Delhi, India). Radioisotopes were procured from Board of Radiation and Isotope Technology (Mumbai, India). All animal experiments in the study were in accordance with the guidelines published by Institute for laboratory animal research and performed after due approval from institute animal ethics committee and CPCSEA, India.

### Expression constructs

The prokaryotic expression constructs of NRL were generated in pGex4T-2 plasmid (GE Life sciences, CT). The human NRL cDNA (AS321 [2]; ) was digested with *Xcm*I and *Hind*III to release the DNA fragment containing the entire coding sequence of NRL. The fragment was treated with T4-DNA polymerase and cloned at *Sma* I restriction site of pGex4T-2 plasmid DNA. Various NRL-deletion constructs were produced by cloning PCR-amplified NRL fragments that incorporated *Eco*RI or *Sma*I restriction sites into pGex-4T-2 expression plasmid, which was digested with appropriate restriction enzymes. Site-directed mutagenesis was used to generate different mutations in the NRL (AS321, cDNA). The primer sets used to generate different mutations in NRL are as follows:

NRL-S50A-sense 5'-GTG CCT CCT GCA CCC ACC TTC AG-3' and antisense 5'-GGT GGG TGC AGG AGG CAC TGA GCT G-3', NRL-P51L-sense 5'-GTG CCT CCT TCA CTC ACC TTC AG-3' and antisense: 5'-GGT GAG TGA AGG AGG CAC TGA GCT G-3', NRL-G122E-sense 5'-GAG ACA GAA GCC CAG CAC GTC-3' and antisense 5'-GGG CTT CTG TCT CCT CTG G-3'. The sequences of all expression constructs were verified before undertaking any protein expression studies. The pEDNRL and pEDNRL-S50T expression constructs have been used previously [11]. Other constructs (pBR130, pcDNA-NRL, and pBCRX) used in the promoter activation assays are described elsewhere [6,11,15].

### Expression and purification of GST-fusion protein in *E. coli*

The full-length, truncated or mutant NRL protein was expressed as a fusion protein with GST in *E. coli* (BL21 strain). GST-fusion protein was purified using glutathione-Sepharose affinity chromatography, as described [18].

### Transfection and metabolic labeling of the protein

COS-1 cells were transfected with a NRL-expression construct (7 mg) using DEAE-dextran method of transfection [3]. After 48 h, the transfected cells were incubated with methionine/phosphate-deprived media for an hour, before substituting with ^35^S-methionine (200 mCi/ml) or ^33^P-orthophosphate (75 mCi/ml) containing media for additional 3 h. Finally, radiolabeled cells were washed three times in cold PBS, containing 1 mM Na-orthovanadate, 1 mM NaF and protease inhibitors. Cells were solubilized in radio-immunoprecipitation assay buffer and used for immunoprecipitation assays. The radiolabeled proteins were precipitated with specific antibodies, analyzed by SDS-PAGE, and visualized by autoradiography.

### Kinase-dependent phosphorylation assay

Affinity-purified GST-NRL or mutant GST-fusion proteins were used to perform activated kinase-dependent phosphorylation assays, in vitro [18,19]. The bead-bound GST-fusion protein (4 mg) was re-suspended in MAPK reaction buffer (50 mM Tris, pH 7.5, 10 mM MgCl_2_, 1 mM EGTA and 2 mM dithiothreitol), and the reaction mixture was incubated for an hour at 30 °C with 0.1 mCi of g^32^P-ATP (>6000 Ci/mmole) and 10 units of purified activated MAPKs (MAPK2 or P38a). The bead-bound proteins were washed three times in 10 mM Tris buffer, pH 7.5, containing 1 mM NaF and 1 mM sodium orthovanadate and solubilized in 1X SDS-lysis buffer by heating for 3 min at 100 °C. The radiolabeled proteins were analyzed by SDS-PAGE, followed by autoradiography. The radioactivity was quantified by exposing gels to phosphor-imager screen, which was scanned using Typhoon phosphor-imager scanner (GE health sciences, Fairfield, CT). The specific activity of the radiolabeled protein was estimated by normalizing the intensity of radioactivity with the protein intensity, as measured with Coomassie brilliant blue.

### Immunoblot analysis

Whole mouse retina was sonicated in 10 mM Tris buffer, pH 7.5 containing protease inhibitors. Retinal lysates (40 mg/lane) were separated on a 12% SDS-PAGE for immunoblot analysis, as described [3].

### Promoter activation assay

To study the transactivation function of NRL, we assayed the activity of rhodopsin promoter in CV-1 cells by co-transfecting pBR130-luc plasmid (luciferase reporter gene under the control of bovine rhodopsin promoter) with pCDNA-NRL, pBCRX, and pCMV-LacZ constructs, as reported previously [11]. The transfection cocktail was prepared by mixing 150 ml of OptiMEM (Invitrogen) with different plasmid DNAs (total 4.5 mg) to equal volume of OptiMEM containing lipofectamine (4 ml/150 ml OprtiMEM) at room temperature for 20 min. The transfection cocktail was further diluted in OptiMEM, dispensed equally in three separate cell culture wells and incubated for 5 h at 37 °C. The transfection cocktail was then substituted with complete DMEM and incubated for additional 24 h under identical cell culture conditions. At 36 h post-transfection, cells were incubated with different MAPK kinase inhibitors (10-20 mmol) for 12 h. The cells were then collected and solubilized in appropriate lysis buffer for luciferase assays. Student's *t*-test was used to evaluate statistical significance between inhibitor-treated and untreated samples.

## Results

### Retinopathy-associated mutations alter metabolic phosphorylation of neural retina leucine-zipper in transfected COS-1 cells

**Figure 1:**
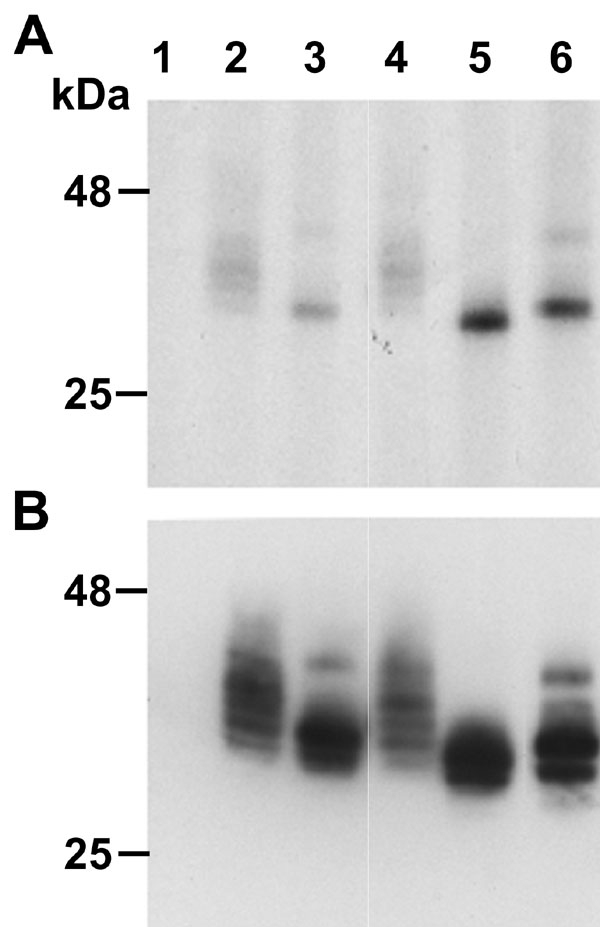
Mutation alters metabolic phosphorylation of neural retina leucine-zipper in transfected COS-1 cells. COS-1 cells transfected with different expression plasmids were radiolabeled with either (**A**), ^33^P-Orthophosphate; or (**B**), ^35^S-Methionine in vitro. COS cell lysates transfected with: Lane 1, pED; lane 2, pEDNRL; lane 3, pEDNRL-S50T; lane 4, pEDNRL-G122E; lane 5, pEDNRL-P51L; lane 6, pEDNRL-S50A were immunoprecipitated using anti-NRL antibody and used equal amount of the total protein to analyze by SDS-PAGE followed by autoradiography. In the figure, NRL-G122E observed to be a non-pathogenic, sporadic mutation in the affected patient was used as a second control in the analysis.

Ser-50 and Pro-51 mutations are shown to alter NRL phosphorylation and NRL-mediated transactivation of rhodopsin minimal promoter in vitro [11,15]. However, direct evidence supporting mutation-induced changes in NRL phosphorylation has not been reported. We therefore performed metabolic phosphorylation of wild-type and mutant NRL in transfected COS-1 cells. Labeled NRL protein was immunoprecipitated and analyzed by SDS-PAGE, followed by autoradiography (Figure 1). Only two major radiolabeled bands were detected for NRL-S50T, -S50A, and -P51L compared to more than six bands for the control NRL and NRL-G122E (a non-pathogenic variant; Figure 1). However, the amount of radioactivity detected for NRL-S50T, -S50A, and -P51L was much higher than the radiolabeled bands observed in control. To examine any possible adverse effect of the mutations on NRL expression, NRL constructs were also transfected in COS-1 cells in presence of ^35^S-methionine. Analysis of the radiolabeled proteins showed that the cumulative intensity of ^35^S-labeled proteins obtained with control and mutant NRL constructs (except NRL-G122E) was fairly similar (Figure 1B). However, the specific bands of 28-30 kDa identified in NRL-S50T, -S50A and -P51L had enhanced ^35^S-methionine labeling compared to corresponding bands (of 28-30 kDa) in control. The mutant NRL protein bands (of 28-30 kDa) that showed enhanced incorporation of ^35^S-methionine also produced an equivalent increase in ^33^P incorporation in the metabolic phosphorylation studies (Figure 1A,B).

### Neural retina leucine-zipper-mutations associated with autosomal dominant retinitis pigmentosa alter mitogen-activated protein kinases-dependent phosphorylation

**Figure 2:**
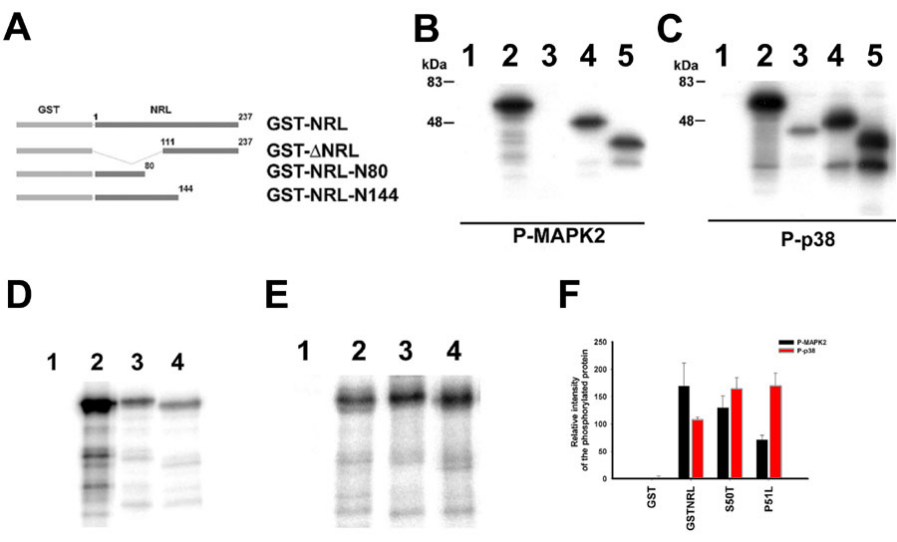
MAPKs produce differential phosphorylation of neural retina leucine-zipper and mutated neural retina leucine-zipper in vitro. **A**: Schematic representation of full length and truncated NRL expressed as GST-fusion protein and used as substrate in the phosphorylation assays. GST-NRL represents the fusion protein expressing the full length NRL; GST-DNRL, with carboxyl terminal 110 amino acids; GST-NRL-N144, with amino terminal 144 amino acids and GST-NRL-N80, with amino terminal 80 amino acids of NRL, respectively. Kinase-dependent phosphorylation assays were performed to phosphorylate full length and truncated GST-NRL using either (**B**) purified activated MAPK2 or (**C**) activated p38a in vitro. Radiolabeled proteins: Lane 1, GST; lane 2, GST-NRL; lane 3, GST-DNRL; lane 4, GST-NRL-N144; lane 5, GST-NRL-N80 were analyzed by SDS-PAGE followed by autoradiography. In similar studies, either (**D**) activated MAPK2 or (**E**) activated p38a was used to phosphorylate GST-NRL with or without specific mutations in vitro. Radiolabed samples analyzed by SDS-PAGE followed by autoradiography includes: Lane1, GST; lane 2, GST-NRL; lane 3, GST-NRL-S50T, lane 4, GST-NRL-P51L in the phosphorylation assay. The specific radioactivity of the protein was estimated by normalizing radioactivity with intensity of the corresponding protein band stained with Coomassie brilliant blue. (**F**) Three independent experiments phosphorylated using similar conditions were used to estimate the relative changes in the radioactivity of the protein. Error bars represent the standard deviation, n=3 in the histogram.

In order to identify the kinases, full-length and truncated NRL were expressed as GST-fusion proteins (Figure 2A) and used as substrate for purified activated MAPK2 and p38a in kinase-dependent phosphorylation assays, in vitro. Activated MAPK2 produced specific phosphorylation of GST-NRL, GST-NRL-N80 (with amino terminal 80 amino acids of NRL) and GST-NRL-N144 (with amino terminal 144 amino acids of NRL), but failed to phosphorylate GSTDNRL (with carboxyl terminal 110 amino acids of NRL) and GST (Figure 2B). On the other hand, activated p38a was able to specifically phosphorylate full-length and almost all NRL-truncations, including GST-DNRL, in an identical phosphorylation assay in vitro (Figure 2C). These results suggest that phosphorylation sites specific to activated MAPK2 are confined to the amino-terminal half of NRL, whereas activated p38a recognizes multiple phosphorylation sites throughout the NRL protein.

To evaluate whether mutations in NRL affect MAPK-dependent phosphorylation of the protein, we used GST-NRL mutants as substrate for kinase-dependent phosphorylation assays, in vitro. Analysis of the radiolabeled proteins indicated that the phosphorylation induced by activated MAPK2 was decreased in GST-NRL-S50T and -P51L mutants compared to the wild-type control (Figure 2D), whereas activated p38a produced enhanced phosphorylation of the GST-NRL-S50T and -P51L mutants (Figure 2E). Purified GST was used as a negative control for phosphorylation assays (Figure 2B-E). Quantification of the specific radioactivity indicated that activated MAPK2 induced 59% decrease in the phosphorylation of GST-NRL-P51L, in contrast to 57% increase in the phosphorylation by activated p38a compared to respective controls (Figure 2F). Identical changes were observed in case of GST-NRL-S50T, but the level of phosphorylation was intermediate to that observed for GST-NRL-P51L and NRL-controls (Figure 2F). The data suggest that mutations induce differential phosphorylation of the mutant NRL by activated MAPK2 and p38a.

### Higher levels of activated mitogen-activated protein kinase-2 are expressed in early postnatal mouse retina

**Figure 3:**
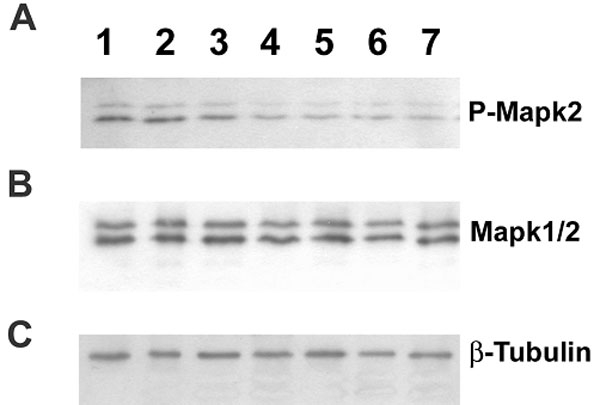
Mitogen-activated protein kinase expression studies in postnatal mouse retina. Postnatal mice retina obtained at: Lane1, (PN) day 0; lane 2, PN 1; lane 3, PN 2; lane 4, PN 3; lane 5, PN 4; lane 6, PN 5, and lane 7, PN 7 were subjected to immunoblot analysis by probing with specific antibodies: **A**, anti-Phospho-MAPK2; **B**, anti-MAPK1/2; and **C**, anti-βTubulin antibodies. The intensity of the immunolabeled bands is directly proportional to the level of specific protein expressed in the retinal samples. Intensity of b-tubulin was used as internal control to normalize the total protein used for analysis.

In mouse retina, multiple phospho-isoforms of NRL are detected as early as postnatal (PN) 2 during development [3]. To determine whether levels of activated MAPK2 expressed in retina coincide with the appearance of NRL phospho-isoforms, total retinal proteins from different postnatal stages of mice were subjected to immunoblot analysis using anti-MAPK antibodies. Enhanced expression of activated (phospho)-MAPK2 was observed in PN 0-3 retina, and this came back to a basal level at and after PN 4 (Figure 3A). However, the levels of MAPK1/2 (un-phosphorylated) expression remained unchanged throughout the entire first postnatal week of retinal development (Figure 3B). All immunoblots were re-probed with anti-b-tubulin antibody as an internal standard to normalize the amount of total retinal protein (Figure 3C).

### Inhibition of mitogen-activated protein kinases signaling affects NRL-mediated transactivation of rhodopsin promoter in vitro

**Figure 4:**
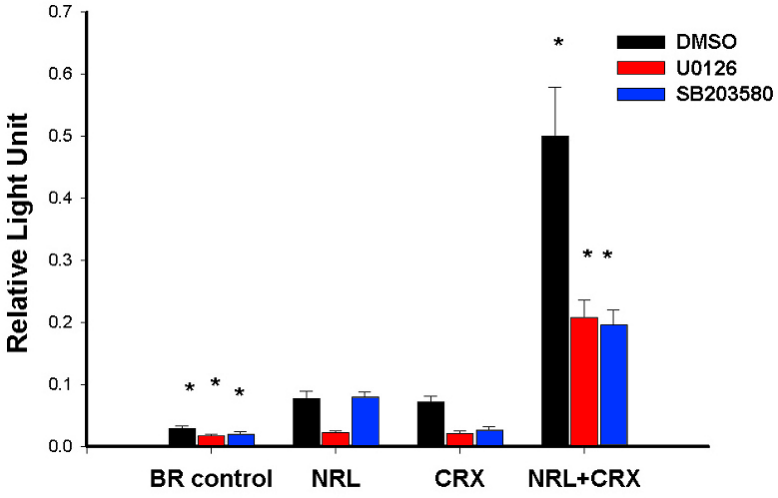
Inhibition of mitogen-activated protein kinase pathways affects neural retina leucine-zipper-mediated transactivation of rhodopsin minimal promoter in transfected CV-1 cells. CV-1 cells were co-transfected with NRL, CRX, or combination of both to transactivate bovine rhodopsin minimal promoter (BR130-luc) in vitro. Luciferase expressed in each reaction condition was normalized with the corresponding amount of b-galactosidase and represented as relative light unit in the histogram. The DMSO treated samples are represented as, black bar and treated as control in the experiment. Whereas, samples treated with kinase inhibitor, U0126 and SB203580 represented as shaded white and shaded brown, respectively. Data from three independent experiments were used to calculate mean and standard error for each experimental condition and students' t-test was used to determine the statistical significance between different treatment groups. Values marked with * represents statistically significant at p<0.001 in the analysis.

The activation of rhodopsin minimal promoter has been used as a functional assay to demonstrate NRL-mediated transcription of retinal genes in vitro [11,15]. To determine the effect of MAPK signaling pathways, COS-1 cells were co-transfected with rhodopsin promoter construct and plasmids expressing different activator proteins (NRL and CRX), and treated with or without specific MAPKs inhibitors, U0126 or SB203580 [20,21]. The amount of luciferase activity was normalized against b-galactosidase to derive the relative light units used as a measure of promoter activity (Figure 4). In cells co-transfected with either NRL or CRX, the treatment with U0126 produced lesser transactivation of the rhodopsin promoter compared to the control (i.e., cells treated with DMSO alone). The cells transfected with NRL alone and treated with SB203580 failed to produce any significant change in the activation of rhodopsin promoter compared to control. However, the cells co-transfected with both NRL and CRX and treated with either U0126 or SB203580 produced significantly less synergistic activation of the rhodopsin proximal promoter compared to the control cells without inhibitors (Figure 4). In a separate experiment, lithium chloride (an inhibitor of GSK3 activation pathway) was used to test whether signaling pathways other than MAPKs produced any significant change in the NRL-mediated transactivation of the rhodopsin promoter (data not shown). Taken together, the results suggest that MAPK-dependent signaling pathways influence NRL-mediated gene regulation in vitro.

## Discussion

Phosphorylation and dephosphorylation of proteins play critical roles in mediating the dynamics of signaling pathways in living cells. NRL is a phospho-protein that performs critical roles during photoreceptor differentiation in mammalian retina [3-5,10]. In this report, we have identified two MAPKs (activated MAPK2 and p38a) that produce specific phosphorylation of NRL, in vitro. Using site-specific mutations and truncated forms of NRL, we demonstrate that a majority of activated MAPK2-dependent phosphorylation sites of NRL are confined to the amino-terminal half of the protein. Activated MAPK can also phosphorylate similar conserved sites in the amino-terminal region of the homologous Maf proteins in vitro [16]. Using separate kinase-dependent phosphorylation studies, we show that adRP-associated S50T and P51L mutations decrease the phosphorylation of mutant NRL protein by activated MAPK2. Interestingly, both of these representative mutations that induce decreased activated MAPK2-mediated phosphorylation are present in the transactivation domain of NRL.

In consensus MAPK-dependent phosphorylation sites, proline is a key residue of the signature sequences [22]. The Pro-51 residue in the transactivation domain of NRL is part of the consensus MAPK-recognition sequence as P51L mutation resulted in reduced phosphorylation by activated MAPK2 in vitro. Our data reveals the role of NRL Pro-51 as an authentic MAPK2-dependent phosphorylation site, which when mutated in retinopathy patients affects the downstream transcriptional regulatory functions of NRL [12-14]. Notably, the mutation at the phospho-amino acid, Ser-50, produces a modest decrease in phosphorylation by activated MAPK2. The phosphorylation studies, reported here, also indicate multiple MAPK sites that contribute to phosphorylation of NRL. We suggest that multiple phospho-isoforms of NRL observed in the retina or in transfected cells are the result of cumulative phosphorylations that are mediated by distinct ubiquitously-expressed kinases [3]. It is also evident that mutations at any single site of NRL are not sufficient to completely eliminate the phosphorylation of the protein.

Both NRL-P51L and -S50T mutations involve a single consensus phosphorylation site in the transactivation domain of NRL. Though in silico analysis did not predict any potential p38a phosphorylation sites in this particular region, both NRL-S50T and P51L mutants produce enhanced phosphorylation by activated p38a in the in vitro assay. We propose that mutations in the transactivation domain of NRL alter the conformation of the protein and expose few uncommon phosphorylation sites, which may otherwise be hidden or less available for active phosphorylation by p38a in the native protein.

The two MAPKs identified here are expressed ubiquitously, consistent with previous reports showing that the profile of NRL phospho-isoforms expressed in COS-1 cells is similar to those detected in the native retina tissue [3,15]. We demonstrate by metabolic phosphorylation studies that mutations in NRL alter cumulative phosphorylation of the protein in vitro. In case of NRL-S50T, -S50A, and -P51L, the partially de-phosphorylated isoforms of NRL are accumulated to form two major bands that show enhanced radiolabeling of both ^33^P and ^35^S-methionine, representing phosphorylation and total protein content, respectively. However, NRL-G122E, which is likely to be a polymorphism fails to produce any significant change in the phosphorylation of the protein Multiple MAPK sites in the transactivation domain of NRL identify it as a phosphorylation hot spot, which is consistent with the prevalence of retinopathy mutations in this region. Similar sites in Maf A and B are also phosphorylated by activated MAPKs [16,17]. Notably, activated p38 is reported to phosphorylate Maf proteins, in vitro [23], and p38-mediated phosphorylation can regulate proliferation and differentiation of neuronal cells in brain [24-26] and cell cycle progression of neuroblastic cells in postnatal rat retina [27].

Our immunoblot analysis showing increased level of activated MAPKs in mouse retina during postnatal day 0-3 is of interest as this is the peak period of rod photoreceptor birth [5,28]. Immunolocalization studies indicate that rod precursors co-express both activated MAPK2 and NRL before the expression of rhodopsin and the lamination and formation of distinct neuronal layers in the retina (data not shown). The higher expression of activated MAPK2 at PN 0-3 is concordant with the reported expression of Nrl phospho-isoforms in vivo [3]. We therefore propose that activated MAPK2 contributes to the phosphorylation of NRL and consequently gene regulation during rod differentiation.

Growth factors promote differentiation and survival of photoreceptors through the activation of MAPK pathways [29]. Identification of NRL as a downstream substrate of MAPK-regulated pathways provides a possible mechanism of how growth factors exert this control. The observation that inhibition of MAPK pathways by treatment with U0126 or SB203580 affects NRL-mediated transactivation of rhodopsin promoter supports this hypothesis. However, decreased transactivation of rhodopsin promoter due to the involvement of signaling cross-talk and role of other potential phospho-proteins within the rhodopsin transcription complex can not be ruled out in current studies.

In conclusion, we have identified NRL as an important substrate of MAPKs. NRL phosphorylation by multiple MAPKs makes it an important cellular target for integrating various signaling pathways to regulate gene expression in developing and mature retina.
